# Challenges with Conventional Dermal Filler Guidelines: Considering Multi-Axes Facial Rotation Asymmetry Patterns

**DOI:** 10.1055/a-2545-1758

**Published:** 2025-05-15

**Authors:** Erik Koppert, Kyu-Ho Yi

**Affiliations:** 1 Department of Surgery, Epworth Hawthorn and Epworth Eastern Private Hospitals, Melbourne, Victoria, Australia; 2 Division in Anatomy and Developmental Biology, Department of Oral Biology, Human Identification Research Institute, BK21 FOUR Project, Yonsei University College of Dentistry, Seodaemun-gu, Seoul, the Republic of Korea; 3 Maylin Clinic (Apgujeong), Seoul, the Republic of Korea

**Keywords:** skilled practitioners, multi-axes facial rotation, facial asymmetry, filler injection

## Abstract

Facial asymmetry is inherent from birth, and it becomes more pronounced with age due to changes in the facial skeleton at various rates and locations. As new insights into “multi-axes facial rotation” patterns emerge, there is a pressing need to update the standards for facial assessment, consultation, and treatment to align with modern aesthetic practices. Traditional methods like MD Codes™ and BeautiPHIcation™, which focus on enhancing specific features or applying mathematical beauty principles, may not adequately address overall facial balance and may neglect the underlying skeletal asymmetries that contribute to a person's appearance. These approaches, while innovative, can result in treatments that might not fully appreciate or correct the foundational asymmetries present in the facial skeleton. Therefore, a comprehensive approach that includes a detailed assessment by skilled practitioners is essential to achieve a balanced aesthetic outcome that not only meets individual aesthetic needs but also enhances patient satisfaction through improved education and trust-building between the clinician and the patient.

Facial asymmetry is an inherent trait present from birth, which becomes more pronounced with age due to changes in the facial skeleton, such as bone resorption, loss of soft tissue volume, and shifts in facial structures. These changes can accentuate preexisting asymmetries, making it crucial for aesthetic practitioners to thoroughly assess each patient's unique needs before recommending treatment. The skull, as any 3D object, allows for rotation around three axes, namely the *x* -, *y* -, and *z* -axes ( Fig. 1 ). New scientific evidence involving a diverse population of 340 individuals has revealed previously unreported common anatomical patterns of “multi-axes facial rotation,” underscoring the need for traditional facial assessment and treatment standards to evolve (“deciphering multi-axis facial rotation: the key to understanding facial asymmetry”—accepted for publication in Plastic and Aesthetic Nursing, Volume 46, Issue 2). Modern aesthetic practices, armed with the knowledge of multi-axes facial rotation patterns, can adapt this latest research to provide state-of-the-art, personalized treatments.

**Fig. 1:**
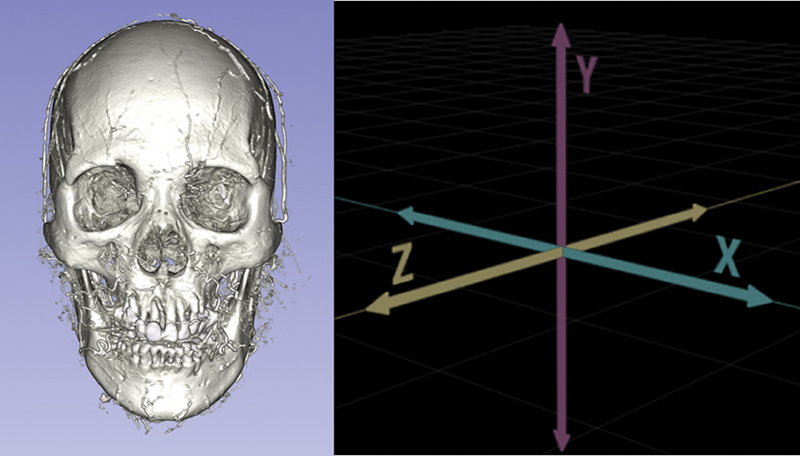
Rotation of the faces can be found on the *x* , *y* , and *z* -axis. This makes it difficult to make generalized injection techniques.

The aim of this communication is to address the limitations of conventional, effective 2D dermal filler guidelines, such as MD Codes™ and BeautiPHIcation™, by not only addressing the complexities of 3D asymmetry but more specifically, particular facial rotation patterns. The conventional methods primarily focus on enhancing individual features rather than achieving overall facial balancing, potentially neglecting broader aesthetic needs.

MD Codes™, developed by Dr. Mauricio de Maio, is a structured approach to dermal filler injections that divides the face into specific zones with precise, symmetrical injection points to achieve targeted aesthetic goals such as lifting, volumizing, and contouring. While MD Codes™ enhance safety and predictability by providing clear guidelines and a standardized, “cooky-cutter” method, they can sometimes overly focus on individual features rather than overall facial balancing. This simplistic approach overlooks broader aesthetic needs, especially in cases of significant multi-axes facial asymmetry patterns or skeletal changes with age. Despite these limitations, MD Codes™ remain a valuable tool to achieve standardized results in facial aesthetics, in particular for novices. 1

BeautiPHIcation™, developed by Dr. Arthur Swift, is a concept that applies the mathematical principles of the “golden ratio” (phi) to facial aesthetics. The golden ratio is a proportion historically associated with beauty, and Dr. Swift's method uses this ratio to guide the enhancement of facial features by pursuing 2D symmetry with dermal fillers. While BeautiPHIcation™ represents a significant advancement in achieving proportionality within individual features, it can also emphasize specific (asymmetrical) proportions at the expense of overall facial balance. By focusing on achieving the golden ratio in isolated facial features, this approach might neglect the broader concept of facial balancing, which would require enhanced individual rotational facial features to be treated to improve overall symmetry and harmony across the entire face. 2

These methods, while structured and scientifically grounded, may not fully address the challenges posed by facial asymmetry and the anatomical changes that occur with aging. As individuals age, the lower face undergoes retrograde movement along the *x* -axis, resulting in volume loss and leading to contractures in the lip depressor muscles and stiffening of the orbicularis oris muscles, causing lip inversion. Notable changes in facial anatomy with age include bone resorption in specific areas of the facial skeleton, such as the pyriform aperture and the maxilla, which deepens the nasolabial crease and causes the nose tip to droop. Additionally, aging affects the mandible, altering its angle and reducing the definition of the lower face, significantly impacting the overall perception of aging. Where asymmetry is present at birth, the asymmetry between the two sides of the face increases steadily with aging (Linden #3857 3 ).

Facial asymmetry often presents along the *x* -axis, where the left side of the face appears more prominent or wider than the right ( Figs. 1 2 3 ). This phenomenon is associated with the “zygion,” the most lateral point of the zygomatic arch, a critical landmark in assessing facial width. Historically, studies have shown that the left side of the face tends to be wider, leading to the term “left face dominance.” (Ercan, 2008 #3859; Hafezi, 2017 #3860). Correcting this asymmetry by widening the right face, as advocated by conventional guidelines is likely to result in poor outcomes and should be planned with great care. Observing the patient from a top–down, or “birds-eye” view helps identify *y* -axis rotational changes (left-to-right, or right-to-left), which can impact facial projection, appearance, and function. With left-to-right rotation, the nose can often be seen deviating to the right. This can be corrected by placing dermal filler in the right piriform fossa and pushing the nose back to the midline. If the injector follows conventional treatment guidelines they may also feel obliged to place dermal filler on the left side, thus maintaining the nasal rotation. Frontal observation of the face allows for the identification of either clockwise or counter-clockwise rotation and associates asymmetries around the *z* -axis. A lower left cheekbone could be separately treated along the superior rim to correct the asymmetry. Such observations are crucial for diagnosing and strategizing suitable interventions for facial asymmetries. The changes in the X, Y, and Z axes with aging and their implications for filler techniques are summarized in Table 1 .

**Fig. 2:**
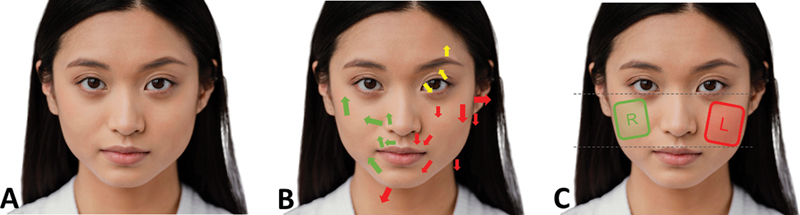
A photograph of an Asian patient with a visibly asymmetrical face. Red arrows indicate the direction of asymmetry, with the left side of the face appearing wider and oriented downward. Green arrows highlight the relative direction of the right side of the face, which appears more lifted and oriented upward compared with the left side.

**Fig. 3:**
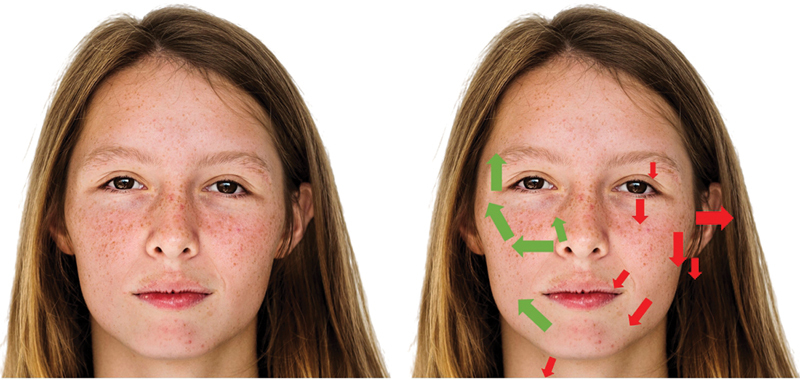
A photograph of a Caucasian patient with a visibly asymmetrical face. Red arrows indicate the direction of asymmetry, with the left side of the face appearing wider and oriented downward. Green arrows highlight the relative direction of the right side of the face, which appears more lifted and oriented upward compared with the left side.

**Table 1:** The effects of aging along the 3D axes of the face

Axis	Aging	Implications for filler
*x* -Axis (horizontal)	Retrograde movement of the lower face; jawline recedes, and chin becomes less prominent. Muscle contractures lead to lip inversion	Focus on restoring volume along the jawline and chin using structural fillers to counteract retrograde changes and muscle contractures
*y* -Axis (vertical)	Vertical shortening of the face; nasal tip droops, and midface volume decreases, affecting overall projection	Enhance midface volume and lift nasal tip with filler placement to improve vertical projection and balance
*z* -Axis (depth/projection)	Asymmetry in facial width and depth; left face dominance becomes more apparent, with one side appearing wider or more prominent	Address asymmetries by carefully adding volume to the narrower or less prominent side to restore overall harmony

To achieve effective treatment outcomes, the manuscript emphasizes the importance of face-to-face consultations and comprehensive assessments by experienced practitioners. Integrating the latest anatomical findings into aesthetic practice is crucial for providing state-of-the-art treatments. By improving individual patient facial assessments and enhancing patient education, practitioners can build trust and credibility, offering clearer and more effective treatment plans that lead to better outcomes and higher patient satisfaction.

The authors advocate for evolving facial assessments, patient education, treatments, and treatment guidelines to reflect the latest discoveries in facial anatomy. This comprehensive approach ensures enhanced patient satisfaction and better aesthetic outcomes, addressing the limitations of contemporary techniques that focus on individual features rather than overall facial harmony (Ercan, 2008 #3862).

In conclusion, while advancements in aesthetic treatments such as MD Codes™ and BeautiPHIcation™ are commendable, it is imperative to incorporate the latest anatomical discoveries for optimal patient outcomes. Current guidelines do not need to be discarded but need to evolve by introducing facial rotation patterns around the *xyz* -axes in their model. The manuscript calls for a more complete, holistic approach that combines the strengths of existing techniques with new insights into facial asymmetry, ultimately leading to more balanced, harmonious, and satisfactory results for patients. 3 4
